# Royal Jelly Protects against Epidermal Stress through Upregulation of the NQO1 Expression

**DOI:** 10.3390/ijms222312973

**Published:** 2021-11-30

**Authors:** Nobuaki Okumura, Takashi Ito, Tomomi Degawa, Mariko Moriyama, Hiroyuki Moriyama

**Affiliations:** 1 Institute for Bee Products and Health Science, Yamada Bee Company Inc., Tomata-gun, Okayama 708-0393, Japan; ti1922@yamada-bee.com (T.I.); ti1740@yamada-bee.com (T.D.); 2 Group Cosmetic Central Laboratory, Yamada Bee Company Inc., Shinagawa, Tokyo 104-0004, Japan; 3 Pharmaceutical Research and Technology Institute, Kindai University, Higashi-Osaka, Osaka 577-8502, Japan; mariko@phar.kindai.ac.jp (M.M.); moriyama@phar.kindai.ac.jp (H.M.)

**Keywords:** royal jelly, fatty acids, keratinocyte, anti-oxidant, NAD(P)H quinone dehydrogenase 1 (NQO1)

## Abstract

Royal jelly (RJ) is secreted by honeybees and has been used as an apitherapy to obtain healthy skin since ancient times. However, the mechanism of the protective effects of RJ against skin aging and skin diseases caused by skin stress and its components have not been clarified. In this study, we attempted to understand the effect of RJ on epidermal function and observed that NAD(P)H quinone dehydrogenase 1 (NQO1) is significantly induced by RJ in keratinocytes. The expression of NQO1 was also increased in the 3D epidermal skin model. NQO1 is involved in antioxidation and detoxification metabolism, and we found that RJ protects against the epidermal stress caused by UVB and menadione through the upregulation of NQO1. We identified 10-hydroxy-2-decenoic acid (10H2DA), a major fatty acid in RJ, as an active compound in this reaction as it induced the expression of NQO1 and protected the skin against oxidative stress. We demonstrated that the protective effect of RJ against epidermal stress is mediated through the upregulation of NQO1 by 10H2DA.

## 1. Introduction

The skin protects us from the outside environment and is continuously exposed to various stresses, such as ultraviolet radiation, air pollution, and pathogens. The epidermis is the outermost tissue directly exposed to these stresses. The functions of the epidermis include a barrier function, moisturizing function, and immune function. It has been reported that the ability to perform these functions declines with age [1,2]. Skin aging is classified as endogenous aging, also known as chronological aging, and exogenous aging affected by UV, air pollution, alcoholism, and nutritional imbalance [3]. The major characteristics of aging skin are thinning and reduced turnover of the epidermis, caused by a delayed recovery of skin damage [4]. Food and cosmetic products have been used to maintain skin health and prevent aging [5,6,7].

Honeybee products such as honey, propolis, royal jelly, and beeswax have been used as apitherapy since ancient times [8]. It has been said that beekeepers have more beautiful hands than other agricultural workers because they are exposed to honeybee products daily. In fact, honeybee products are used as ingredients in medicines and cosmetics for wound healing and moisturizing [9].

Royal jelly (RJ) is a yellowish-white creamy substance secreted by honeybees (*Apis mellifera*). RJ is an essential nutrient for the maturation of bee larvae into queen bees. Therefore, RJ has been used as an anti-aging factor since ancient times [10,11]. Recent clinical studies have demonstrated that RJ ingestion improves skin moisturizing [12,13] and skin pigmentation [12]. In addition, the topical application of RJ is widely used in dermatology and skin care [9]. Topical application of RJ alleviates the pruritus in the allergic contact dermatitis model [14]. It has also been reported that the topical application of RJ promotes the wound healing model. A clinical trial was conducted to evaluate the efficacy of topical royal jelly on healing diabetic foot ulcers [15].

Fresh royal jelly chemically contains water (60–70%), carbohydrates (11–23%), proteins (9–18%), lipids (4–8%), and other low compounds such as vitamins and minerals [16]. Studies exploring the active components of RJ have been conducted. It has been reported that defensin, an antibacterial peptide in RJ, promotes tissue remodeling during wound healing in the mouse epidermis by inducing MMP-9 expression [17], and the major royal jelly protein (MRJP) enhances keratinocyte migration [18]. Furthermore, 10-hydroxy-2-decenoic acid (10H2DA), a unique medium-chain fatty acid in RJ, promotes the differentiation of epidermal keratinocytes and affects the moisturizing function of the epidermis [19]. However, the effect of royal jelly on epidermal stress, a direct cause of skin aging, and its mechanism of action and active components, have not been elucidated.

In this study, we explored the protective effect of RJ against skin stress. We identified that NQO1 was upregulated in primary skin keratinocytes cultured with RJ. As NQO1 is a stress-responsive gene, we investigated the effect of RJ on stress protection of the epidermis and found that 10H2DA in RJ induces the expression of NQO1 and protects the skin from stress.

## 2. Results

**Figure 1:**
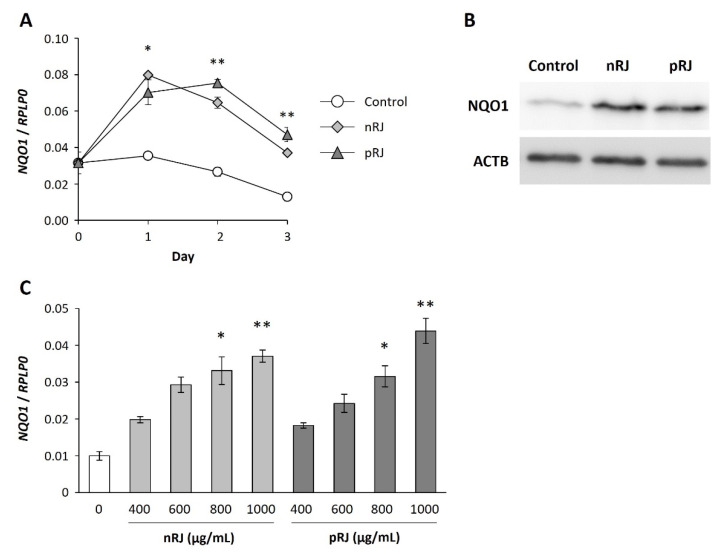
Effect of RJ on NQO1 expression in keratinocytes. (**A**) Time-dependent expression of NQO1 mRNA in NHEK (pooled) following stimulation with either 1 mg/mL nRJ or pRJ. Each point represents the mean ± SEM (*n* = 3–4). * *p* < 0.05, ** *p* < 0.01 vs. control. (**B**) Western blot analysis of NQO1 three days after stimulation with either 1 mg/mL nRJ or pRJ. Representative images from three independent experiments are shown. (**C**) Dose-dependent expression of NQO1 mRNA in NHEK (pooled) following stimulation with either nRJ or pRJ. Each bar represents mean ± SEM (*n* = 3–5). * *p* < 0.05, ** *p* < 0.01 vs. 0 μg/mL.

We first explored the changes in gene expression related to epidermal function following exposure to RJ. A heatmap analysis revealed that lyophilized raw royal jelly (nRJ) treatment induced the expression of some of the genes related to keratinocyte differentiation (Supplemental Figure S1). However, the expression pattern was different from that induced by CaCl_2_, a well-known inducer of epidermal differentiation (Supplemental Figure S1). We analyzed genes whose expression was significantly different (more than two-fold changed) after RJ treatment. NQO1 showed the most significant change in expression. NQO1 is an enzyme that regulates anti-oxidation and immunoreaction in the skin. Therefore, we investigated the change in NQO1 expression after nRJ exposure in detail. NQO1 was upregulated from day one after nRJ stimulation until day 3 (Figure 1A). As NQO1 is downstream of the Nrf2 pathway, we investigated the effect of RJ on other genes downstream of Nrf2. No change in the expression of Homx1 and Txn1 was observed following exposure to RJ, although the expression of Gclc was significantly increased after the first and second day of stimulation with nRJ (Supplemental Figure S2). These results suggest that nRJ induces the expression of NQO1 in keratinocytes. We confirmed that the protein expression of NQO1 in keratinocytes was also upregulated after three days in nRJ-treated keratinocytes compared to that in the control (Figure 1B). We also demonstrated that nRJ induced the expression of NQO1 in a dose-dependent manner (Figure 1C). We confirmed that the induction of NQO1 by nRJ was independent of the keratinocyte lot and nRJ lot (Supplementary Figure S3).

**Figure 2:**
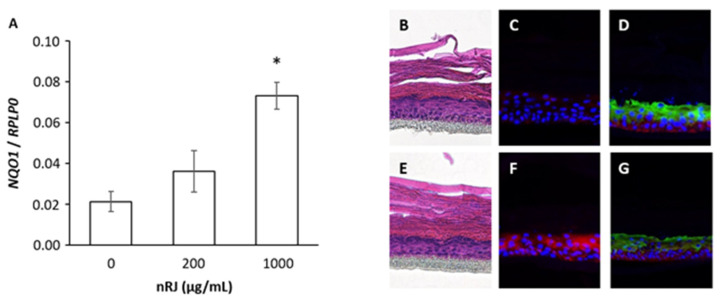
Effect of RJ on the expression of NQO1 in a 3D epidermis model. The 3D epidermis model was established as described in Materials and Methods. Eleven days after starting the 3D epidermis constriction, it was treated with nRJ for three days. (**A**) The expression of NQO1 mRNA three days after stimulation with nRJ. Data represent the mean ± SEM of three independent experiments. * *p* < 0.05. (**B**–**G**), Histological analysis of the effect of RJ on NQO1 expression. Panels (**B**–**D**) are shown for the control. Panels (**E**–**G**) are shown in the 1 mg/mL nRJ treated group. The establishment of the epidermal structure was confirmed by staining with hematoxylin-eosin (**B**,**E**) and the immunoreactivity of anti-keratin14 antibody (for basement membrane, red staining in (**D**,**G**)) and anti-keratin10 antibody for stratum granulosum and stratum spinosum, green staining in (**D**,**G**)). NQO1 was detected using an anti-NQO1 antibody (red staining in (**C**,**F**)). The nuclei were visualized using Hoechst staining.

Furthermore, we analyzed the induction of NQO1 by nRJ using a 3D skin epidermal model. The results showed that the expression level of NQO1 was increased in cells from the basal layer to the granular layer in the 3D skin epidermal model (Figure 2), suggesting that nRJ induces NQO1 expression in the epidermis.

**Figure 3:**
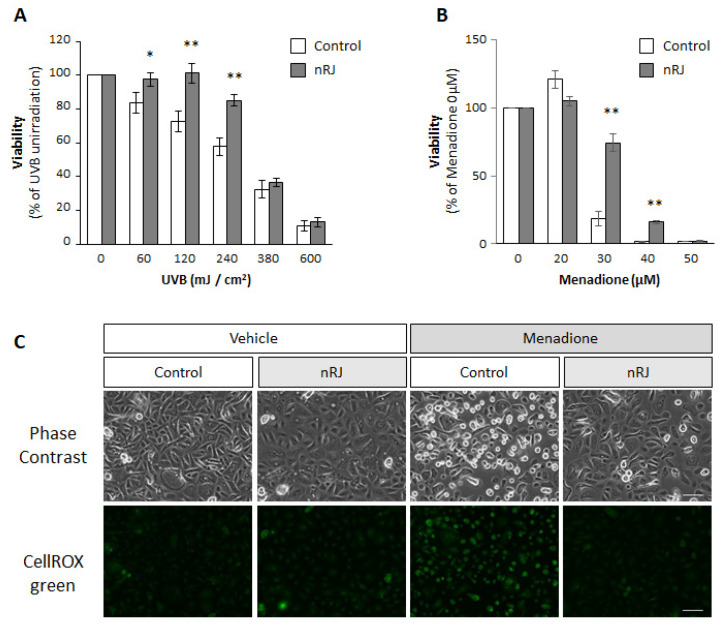
Effect of RJ on the cytotoxicity induced by epidermal stressors in keratinocyte. NHEK (pooled) were treated with 1 mg/mL nRJ for 3 days and then the skin stress was induced as indicated. (**A**) UVB irradiation was carried out at the indicated doses. After 24 h of UVB irradiation, the cell viability was assessed. The value expresses the percentage of UVB irradiation. Data represent the mean ± SEM (*n* = 3). * *p* < 0.05, ** *p* < 0.01 vs. control, Student’s paired *t*-test. (**B**) Menadione treatment was performed with the indicated concentrations. One hour after treatment, the cells were washed with PBS and then cultured in fresh medium for 24 h. The viability was measured. The value expresses the percentage of menadione untreated (0 μM). Data represent the mean ± SEM (*n* = 3). ** *p* < 0.01 vs. control, Student’s paired *t*-test. (**C**) NHEK were pretreated with CellROX green for 30 min and then stimulated with 30 μM menadione for 1 h. The upper panel shows the phase contrast, and the lower panel shows CellROX green. Representative images from three independent experiments are shown. Scale bar indicates 100 μm.

Next, we attempted to understand the functional significance of the induction of the NQO1 expression by RJ in the epidermis. As NQO1 is an enzyme involved in anti-oxidation and detoxification [20], we investigated the protective effects of RJ against skin stressors, such as UVB irradiation and oxidative stress. Cytotoxicity was observed in a dose-dependent manner when keratinocytes were irradiated with UVB (Figure 3A). In contrast, cells treated with nRJ showed lower UVB-induced cytotoxicity (Figure 3A). Next, we examined the effects of RJ on menadione-induced cytotoxicity. We used menadione, an oxidative stress inducer, as a quinone model, an air pollutant [21,22,23]. We confirmed dose-dependent keratinocyte cytotoxicity following exposure to menadione. In contrast, a significant protective effect against menadione-induced cytotoxicity was observed in the nRJ-treated keratinocytes (Figure 3B). To check whether the suppression of reactive oxygen species mediated this cytoprotective effect, we analyzed the level of oxidative stress after menadione treatment using CellROX green. We observed that treatment with RJ suppressed menadione-induced morphological changes in keratinocytes and oxidative stress, as indicated by CellROX green (Figure 3C). These results suggest that RJ has a protective effect on the epidermis of the skin by preventing oxidative stress.

**Figure 4:**
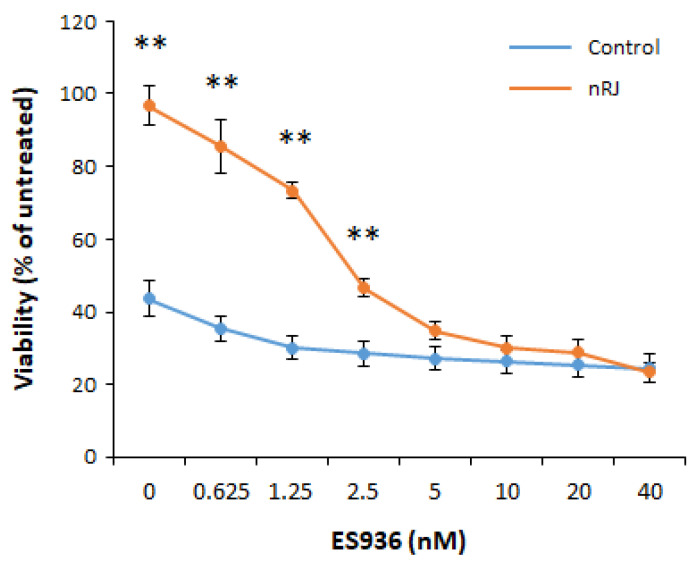
Effect of the NQO1 inhibitor, ES936, on the menadione-induced cytotoxicity in keratinocytes. NHEK (23 y) were treated with 1 mg/mL nRJ for three days. NHEK were pretreated with ES936 for 30 min then exposed to 30 μM menadione for 1 h, then the cells were washed with PBS and cultured in the fresh medium for 24 h. The viability was measured with WST-8. The value expressed the percentage of neither the vehicle nor ES936 untreated cells. Data represent mean ± SEM (*n* = 4). ** *p* < 0.01 vs. control, Student’s paired *t*-test.

Next, we examined whether the cytoprotective effect against menadione was mediated by the increased expression of NQO1 induced by RJ using an NQO1 inhibitor, ES936, which has been reported to inhibit NQO1 activity at the cellular level [24]. The treatment of keratinocytes with ES936 for 1 h before menadione treatment decreased the cytoprotective effect of nRJ in a dose-dependent manner (Figure 4). ES936 treatment at 10 nM or higher reduced the RJ-mediated cytoprotective effect to the same level as the control (Figure 4). This result indicated that the increased expression of NQO1 by nRJ contributed to the protective effect against menadione-induced cytotoxicity.

**Figure 5:**
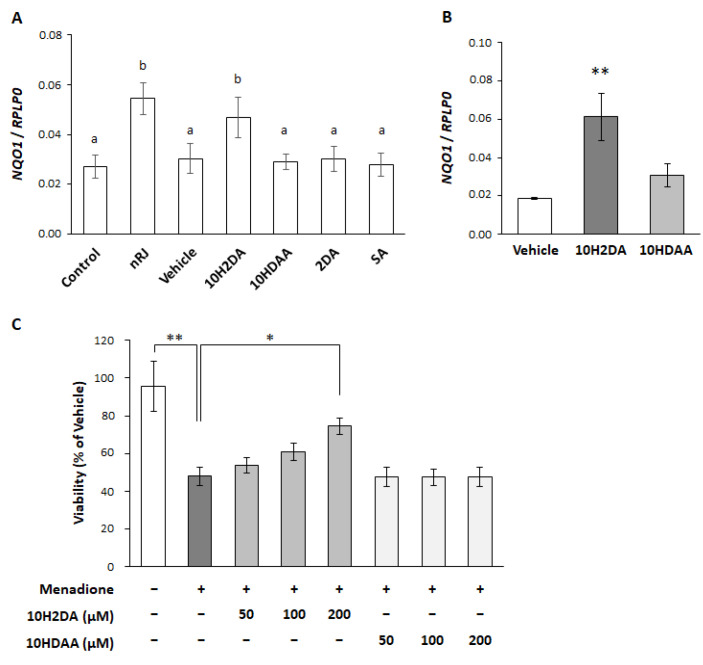
Effect of 10H2DA and 10HDAA on the expression of NQO1 and menadione induced cytotoxicity. (**A**) Screening of RJ-specific fatty acids responsible for inducing the NQO1 expression. Keratinocytes (23 y) were treated with 200 μM of each fatty acid for 1 d. The NQO1 mRNA expression was analyzed by real-time PCR. Data represent the mean ± SEM (*n* = 4). The different letters (a, b) above the bars indicate statistical difference. (**B**) The expression of NQO1 in NHEK (pooled) following stimulation with 200 μM 10H2DA and 10HDAA for 3 days. Data represent mean ± SEM (*n* = 5). ** *p* < 0.01. (**C**) The effect of 10H2DA and 10HDAA on menadione-induced cytotoxicity. NHEK (23 y) were treated with either 10H2DA or 10HDAA for three days. After that, NHEK was treated with 30 μM menadione for 1 h, then, the cells were washed with PBS and cultured in the fresh medium for 24 h. The viability was measured with WST-8. The value expresses the percent of vehicle. Data represents mean ± SE (*n* = 4). * *p* < 0.05, ** *p* < 0.01 vs. control.

Next, we tried to identify the factors in RJ which induce NQO1 expression. RJ is a mixture of various components, including proteins and lipids. Since it has been reported that peptides in RJ, such as defensin-1 and MRJP, are involved in keratinocyte migration and wound healing, we first investigated whether protease-hydrolyzed RJ (pRJ) [25] increases NQO1 expression. The pRJ showed temporal and concentration-dependent NQO1 expression and protein induction activities (Figure 1A–C), suggesting that the induction of NQO1 expression is not dependent on a protein component in RJ. Therefore, we investigated the effects of 10H2DA and 10-hydroxydecanoic acid (10HDAA), which are medium-chain fatty acids characteristic of royal jelly, and their dicarboxylic acids, decenedioic acid (2DA), and sebacic acid (SA) [26] on the expression of NQO1. We observed that only 10H2DA induced NQO1 mRNA expression one day after stimulation with RJ (Figure 5A). We compared the effects of 10H2DA and 10HDAA on the expression of NQO1 expression and their cytoprotective activities. 10H2DA significantly induced NQO1 expression three days after stimulation, whereas 10HDAA did not show induce NQO1 upregulation (Figure 5B). We further compared the effects of 10H2DA and 10HDAA on menadione-induced keratinocyte toxicity (Figure 5C). We observed a concentration-dependent cytoprotective effect in the presence of 10H2DA. However, no cytoprotective effect was observed in the presence of 10HDAA (Figure 5C). These results suggested that 10H2DA was the active compound showing the cytoprotective effect of RJ through upregulation of NQO1.

## 3. Discussion

In this study, we found that NQO1 expression was increased by 10H2DA in normal human epidermal keratinocytes and its 3D epidermal model. Furthermore, we demonstrated that the upregulation of NQO1 by RJ exerts protective effects against skin stresses, such as UV irradiation and menadione-induced oxidative stress. These results indicate that the induction of NQO1 by RJ has a protective effect on the epidermis. NQO1 is the major quinone reductase and has a superoxide reductase activity that regulates detoxification and redox metabolism [20]. In *NQO1* knockout mice, increased toxicity against menadione was reported [27]. It has also been reported that *NQO1* knockout mice showed sensitivity to carcinogens in the skin resulting in cancer [27,28]. The induction of the NQO1 gene expression has been reported to show protective effects against UV stimulation and environmental pollution on the skin [29,30] in cooperation with other antioxidant genes. This skin protective effect of RJ found in this study will need to be further investigated in clinical trials.

In this study, we found that 10H2DA was a promising candidate involved in the increase in NQO1 expression in RJ and its association with the protective effect of keratinocytes. To clarify the active compound, we first investigated the involvement of the proteins and peptides in RJ in inducing NQO1 expression. Although we used pRJ, in which proteins were degraded by alkaline peptidase [25], no difference was observed in the induction of NQO1 expression compared to nRJ (Figure 1). Therefore, we focused on the fatty acids present in RJ. It has been reported that RJ contains approximately 4.2% 10H2DA and approximately 1.5% 10HDAA, which are unique medium-chain fatty acids. These fatty acids are not affected by alkaline peptidase treatment [26]. In this study, the maximum concentration of RJ used in the culture was 1 mg/mL. The concentration of 10H2DA, the most abundant fatty acid in RJ, in 1 mg/mL of RJ was calculated to be approximately 230 μM. Therefore, we treated keratinocytes with 200 μM of each fatty acid (10H2DA, 10HDAAA, 2DA, and SA). We found that only 10H2DA induced the expression of NQO1. Furthermore, after comparing the results of the NQO1 gene expression and the cytoprotective effects of the fatty acids in RJ, 10H2DA was found to be the primary active component. However, other traceable compounds including, C8, C10, and C12 fatty acids, have been found in RJ by gas chromatography-mass spectrometry [31]. To fully explain the effect of RJ on NQO1 expression, we need to explore the suitable combination of RJ fatty acids.

In this study, we found that 10H2DA induced NQO1 expression, but 10HDAA did not. Several studies have been reported on fatty acids in RJ that exert various physiological functions. For example, 10H2DA and 10HDAA have been reported to have an agonist activity for TRPA1, a receptor involved in heat production and energy expenditure [32]. Recently, it was reported that 10H2DA has an agonist activity against FFAR4, a known fatty acid receptor [33], but there are no reports on whether 10HDAA binds FFAR4. 10H2DA and 10HDAA have been shown to bind specifically to ERβ [34]. In addition, other RJ fatty acids, such as 3,10DDA, SA, and 10H2DA, have been reported to regulate the recruitment of ERα and ERβ to the binding region of their target genes during transcription [35]. As these effects differ in the presence and absence of estrogen, analyzing the effects of fatty acids on estrogen receptor modulation is not straightforward. We have also recently reported that 10H2DA, 10HDAA, 2DA, and SA all regulated the myogenesis of C2C12 cells, murine myoblast cells [36]. In addition, the clear differences in the activity of different types of fatty acids in RJ are not reported. In this study, we found a clear difference in the induction of NQO1 by 10H2DA and other major RJ fatty acids (10HDAA, 2DAA, and SA). These results suggest that the induction of the NQO1 expression by 10H2DA in keratinocytes might be different from the receptor-mediated mechanism of the action reported so far. Further studies are required to address these points.

The induction of NQO1 expression in the epidermis has been reported to be mediated by the AhR and Nrf2 pathways [37,38,39], and, likely, the induction of the NQO1 expression by RJ and 10H2DA is also mediated through these pathways. In a study on fatty acids in *Coriandrum sativum* L., Abiko et al. reported that covalent modification by the 2-alkenyl group of aliphatic electrophiles activated Nrf2/Keap1 and increased the expression of Gclm and HO-1, which are downstream of Nrf2 in HepG2, in a liver cancer cell line. In the present study, 10H2DA with 2-alkenyl modification induced the NQO1 expression, while 10HDAA, 2DA, and SA showed no effects, suggesting that 10H2DA induces NQO1 via the Nrf2 pathway. We also revealed that 2DA, dicarboxylic acid with 2-alkenyl did not induce NQO1 mRNA (Figure 5A). The structural difference between 10H2DA and 2DA is the terminal functional group. Although the terminal functional group of 10H2DA is the hydroxyl group, the terminal group of 2DA is the carboxyl group. The terminal functional group is known to affect membrane permeability. For instance, Log P is one of the indicators of membrane permeability. The estimated Log P value of 10H2DA and 2DA in the PubChem database is 2.2 and 2.0, respectively. These results suggested that 2DA is less membrane-permeable than 10H2DA. Further studies are required about the structural−activity relationship among RJ fatty acids.

On the other hand, as RJ did not induce the expression of HO-1 and Txn, which are representative genes downstream of Nrf2, RJ might induce NQO1 expression via other unknown mechanisms. The AhR-mediated pathway has also been reported to induce NQO1 expression in the skin. For example, coal tar, used as a treatment for atopic dermatitis, has been reported to increase the expression of NQO1 and Filaggrin via AhR [37]. In the present study, we revealed that RJ significantly increased both Flg and NQO1 (Supplemental Figure S1). These results suggest that RJ might exert its effects via the AhR pathway. The molecular mechanism that triggers the induction of the epidermal NQO1 expression by RJ and 10H2DA needs to be investigated further in future studies.

RJ has been used in skincare as an apitherapy agent. However, the molecular mechanism underlying the benefits provided by RJ are not yet fully understood. In this study, we showed that RJ protects against epidermal stressors, such as UVB and oxidative stress inducers. As UVB and oxidative stress are direct causes of epidermal aging, RJ is considered useful for skin aging protection.

## 4. Materials and Methods

Lyophilized raw royal jelly (nRJ) and lyophilized protease-treated royal jelly (pRJ) were prepared by Yamada Bee Company, Inc. (Okayama, Japan). RJ was standardized with the amounts of specific fatty acids (E)-10-hydroxy-2-decenoic acid (10H2DA) and 10-hydroxydecanoic acid (10HDAAA): nRJ contained a minimum of 3.8% of 10H2DA and a minimum of 0.6% of 10HDAA, while pRJ contained a minimum 3.5% of 10H2DA and a minimum 0.6% of 10HDAA. 10H2DA was purchased from Hangzhou Eastbiopharm, Co., Ltd. (Hangzhou, China), and 10HDAA was purchased from Combi-Blocks, Inc. (San Diego, CA, USA). 2-Decenedioic acid (2DA) was purchased from Sundia MediTech Co., Ltd. (Shanghai, China), and sebacic acid (SA) was purchased from Sigma-Aldrich Co., LLC. (St. Louis, MO, USA). The antibodies used in this study are listed in Supplementary Table S1.

### 4.1. Normal Human Epidermal Keratinocyte Culture

The normal human epidermal keratinocytes (NHEK) used in this study are shown in the Supplementary Table S2. NHEK cells were maintained in keratinocyte growth medium 2 (KGM2, Promocell, Heidelberg, Germany) and used under nine passages. The cultured NHEKs were detached with TrypLE™Express Enzyme (Thermo Fisher Scientific, Waltham, MA, USA) and then inoculated at a density of 3 × 10^4^ cells/cm^2^. nRJ (Lot YDP-M-180120, YDP-M-190801, and YDP-M-200225) and pRJ (Lot YRP-M-181030) were suspended in KGM2 at 1 mg/mL and then extracted by sonication for 30 min. The concentration of 10H2DA in royal jelly using in this study was described in Supplemental Table S3. Before adding it to the culture, the RJ solution was filtrated through a 0.20 µm filter (DISMIC-25, Advantec, Tokyo, Japan). Royal jelly fatty acids 10H2DA, 10HDAA, SA, and 2DA were dissolved in DMSO to make 200 mM solutions and were diluted to 200 μM in KGM2 before adding it to the culture. Two millimoles of CaCl_2_ were used to differentiate the keratinocytes.

### 4.2. 3D Epidermis Keratinocyte Culture

A 3D epidermal keratinocyte culture was performed using the CnT-3D Epithelial Starter Kit (Cellntec, Bern, Switzerland) and according to the manufacturer’s protocol. Eleven days after the initiation of 3D culture, nRJ was added to the medium and cultured for another three days. The samples were then harvested and used for analysis.

### 4.3. Quantification of mRNA Expression Using qRT-PCR

Total RNA was extracted from NHEKs using the NucleoSpin^®^ RNA Plus kit (Takara, Shiga, Japan). The concentration of total RNA was determined using Nanodrop One (Thermo Fisher Scientific). The total RNA (500 ng) was reverse transcribed using the ReverTra Ace^®^ qPCR RT Master Mix (Toyobo, Osaka, Japan). Real-time polymerase chain reaction (PCR) was performed using the SYBR Green method with SsoAdvanced™ Universal SYBR^®^ Green Supermix (Bio-Rad Laboratories, Inc., Hercules, CA, USA) and a CFX Opus real-time PCR system (Bio-Rad Laboratories). The primer pairs used in this experiment are listed in Supplementary Table S4. We selected Rplp0 as a housekeeping gene from 15 human housekeeping gene primer sets (Takara) after evaluating the stability using CFX Maestro™ software (Bio-Rad Laboratories Inc.).

### 4.4. Stress Induction in Keratinocytes

We used a UVB irradiation model and a menadione-induced oxidative stress model. Stress induction was performed after exposing NHEK to RJ for three days. In the UVB irradiation model, NHEK cells were washed with PBS and irradiated by using a UV transilluminator (95-0343-01, Analytik Jena AG, Jena, Germany). The intensity of UVB was monitored using a UV meter (UV-340A, Kenis, Tokyo, Japan). After irradiation, the medium was replaced with fresh KGM2 containing RealTime-Glo™ MT Cell Viability Assay (Promega, Madison, WI, USA) components. Luminescence was measured after 24 h using an Envision plate reader (PerkinElmer, Waltham, MA, USA).

NHEK cells were treated with menadione (Sigma-Aldrich) for 1 h and then washed with PBS for the menadione-induced oxidative stress model. The NHEK cells were further cultured in KGM2 containing RealTime-Glo™ MT Cell Viability Assay (Promega) components. Luminescence was measured after 24 h using an Envision plate reader (PerkinElmer). For evaluating the NQO1 activity in menadione-stimulated oxidative stress, the NQO1 inhibitor, ES936, was added 30 min before menadione treatment. Viability was measured using a WST-8 based assay with Cell Count Reagent SF (Nacalai Tesque, Kyoto, Japan).

### 4.5. Detection of Oxidative Stress

Keratinocytes were plated on cell imaging slides (Eppendorf, Hamburg, Germany) at a density of 6 × 10^4^ cells/cm^2^ and cultured for 24 h. Next, the medium was replaced with KGM2 containing 1 mg/mL nRJ. After three days of cultivation, CellROX^®^ Green (Thermo) was added to the culture at a concentration of 5 μM and was incubated for 30 min. Cells were washed twice with Live Cell Imaging Solution (Thermo) and then incubated with KGM containing 30 μM menadione to induce oxidative stress for 1 h. After menadione stimulation, the cells were washed twice with Live cell imaging solution, fixed with 4% paraformaldehyde (Nacalai Tesque) for 10 min, and then overlayed with Fluoromaunt G. Images were obtained using BZ-X800 (Keyence, Tokyo, Japan).

### 4.6. Western Blot Analysis

Total cellular protein was extracted in RIPA buffer (Nacalai Tesque) containing a protease inhibitor cocktail (Nacalai Tesque). The protein concentration was determined using a BCA Protein Assay Kit (Thermo Fisher Scientific). Ten micrograms of the total protein extract were subjected to SDS–PAGE. After electrophoresis, the proteins were transferred onto a PVDF membrane using the Trans-Blot Turbo transfer system (Bio-Rad). Immunoreaction was performed using Immobilon^®^ GO (Millipore, Billerica, MA, USA) according to the manufacturer’s protocol. Anti NQO1 antibody (Cell Signaling, Danvers, MA, USA) and HRP-conjugated anti-rabbit IgG were used in the immunoreaction. The protein bands were visualized using Western Lightning Clarity Western ECL Substrate (Bio-Rad). The intensity of each band was quantified using an image analyzer (LAS-2000, Fujifilm, Tokyo, Japan).

### 4.7. Histological Analysis

The 3D epidermal culture was fixed with a cold 4% paraformaldehyde (Nacalai Tesque) solution overnight, and the ethanol was replaced with G-NOX (GenoStaff, Tokyo, Japan). Afterward, the cultures were paraffin-embedded. Sections (10 μm thickness) were prepared using a microtome. The epidermal integrity was confirmed after staining with hematoxylin-eosin and the anti keratin14 antibody (for basement membrane) and anti keratin10 antibody for stratum granulosum and stratum spinosum. The expression of NQO1 was analyzed using an NQO1 antibody. Briefly, the prepared sections were blocked with TNGS (0.3% Triton X-100, 1% normal goat serum) at room temperature for 1 h. Subsequently, an anti-NQO1 primary antibody (mouse; 1:400), an anti-KRT10 primary antibody (rabbit; 1:250), and an anti-KRT14 primary antibody (mouse; 1:1000) were incubated with the sections overnight at 4 °C. The sections were washed with T-PBS and incubated with goat anti-mouse IgG 555 (1:2000) and anti-rabbit IgG 488 (1:2000) for 1 h at room temperature. After washing with T-PBS, the sections were stained with DAPI-Fluoromount-G (Southern Biotech, Birmingham, AL, USA). Images were obtained using a BZ-X800 microscope (Keyence).

### 4.8. Statistical Analysis

All of the statistical analyses were performed using Excel and GraphPad Prism7. The experimental replicate number is demonstrated in the figure legends. The data are represented as mean ± standard error of the mean (SEM). The statistical differences among groups were analyzed by the non-parametric Kruskal–Wallis test (post-hoc Dunn’s multiple comparisons test) or parametric one-way analysis of variance (post-hoc Tukey’s or Dunett’s multiple comparisons test). If two groups were analyzed, the significance was determined by the Mann–Whitney’s U test (non-parametric) or Student’s *t*-test (parametric). Statistical significance was set at *p* < 0.05.

## Data Availability

Not applicable.

## Supplementary Materials

The following are available online at www.mdpi.com/article/10.3390/ijms222312973/s1.
