# Supplementary material for: Royal Jelly Protects against Epidermal Stress through Upregulation of the NQO1 Expression
Source: Int J Mol Sci. 2021 Nov 30;22(23):12973. doi: 10.3390/ijms222312973 (PMC8657709; doi:10.3390/ijms222312973)
Supplement: Supplementary file 1 [file ijms-22-12973-s001.zip › ijms-1476870-supplementary.pdf]

Supplemental Table S1. Antibodies used in this study.

Antibodies	Supplier	Cat.	Usage
Nqo1	CST	#3187	Western Blotting
β-Actin	MBL	M177-3	Western Blotting
Keratin 14	abcam	ab7800	Immunohistochemistry
Keratin 10	abcam	ab76318	Immunohistochemistry
Nqo1	CST	#62262	Immunohistochemistry
Anti-mouse IgG, HRP-linked Antibody	CST	#7076	Western Blotting
Anti-mouse IgG (H+L), F(ab')2 Fragment (Alexa Fluor® 555 Conjugate)	CST	#4409	Immunohistochemistry
Anti-rabbit IgG (H+L), F(ab')2 Fragment (Alexa Fluor® 555 Conjugate)	CST	#4413	Immunohistochemistry
Anti-mouse IgG (H+L), F(ab')2 Fragment (Alexa Fluor® 488 Conjugate)	CST	#4408	Immunohistochemistry
Anti-rabbit IgG (H+L), F(ab')2 Fragment (Alexa Fluor® 488 Conjugate)	CST	#4412	Immunohistochemistry

Supplemental Table S2. Normal human epidermal keratinocytes used in this study.

Supplier	Sex	Age	Race	Tissue		Lot number
PromoCell	female<br>female<br>female	31<br>32<br>51	Caucasian<br>Caucasian<br>Caucasian	skin<br>breast<br>eyelid	Pooled	433Z033
PromoCell	female	56	Caucasian	abdomen	Single	451Z0141
BIOPREDIC International	female	23	Caucasian	abdomen	Single	KER110016

Supplemental Table S3. The concentration of 10H2DA in royal jelly.

**Royal jelly**	**Lot**	**10H2DA (%)**	**Method**
Lyophilized raw royal jelly	YDP-M-180120	4.65	HPLC
Lyophilized raw royal jelly	YDP-M-190801	4.86	HPLC
Lyophilized raw royal jelly	YDP-M-200225	4.58	HPLC
Lyophilized protease-treated royal jelly	YRP-M-181030	4.06	HPLC

Supplemental Table S4. Primer pairs used in this study

**Synbol**	**Gene name**	**Forwead, Sequence (5'->3')**	**Reverse, Sequence (5'->4')**
AHR	Aryl hydrocarbon receptor	TGGTTGTGATGCCAAAGGAAGAA	ACTGGACCCAAGTCCATCGG
AQP3	Aquaporin 3	CCGTGACCTTTGCCATGTGCTT	TTGTCGGCGAAGTGCCAGATTG
CAPN1	Calpain-1	TTCGAGCCCAACAAGGAGGG	AGAGCACTTGCTCATCGGGG
CASP14	Caspase-14	CAGCCCACAAACCATCCCAAC	GTAGGCGATGTATCCCTCTACCGT
CAT	Catalase	AATGCTTCAGGGCCGCCTTT	ACTCGAGCACGGTAGGGACA
CD44	CD44 molecule	AGTGGTTTGGCAACAGATGGC	GAGGCTGCAGCTGTCCCTGT
CEMIP	Cell migration inducing hyaluronidase 1	GGAGCCGGCCATCATCAGAC	GGGAAGGTTCCACCACTGGC
CLDN1	Claudin 1	GTCTTTGACTCCTTGCTGAATCTG	CACCTCATCGTCTTCCAAGCAC
COL17A1	Collagen type XVII alpha 1 chain	CAGGACCTGTCACCACCATC	CGTACCCCGAAGTCCGTAAG
COL4A1	Collagen type IV alpha 1 chain	GAGTGCCTGGAAAGGACGGG	GGTGTTCCTGGCAAGCCCAT
CTSH	Cathepsin H	TCATGGATGTCTAAGCACCGT	GGTTCCCATTGTTGTGGGCG
CYP1A1	Cytochrome P450 family 1 subfamily A member 1	GATTGAGCACTGTCAGGAGAAGC	ATGAGGCTCCAGGAGATAGCAG
FN1	Fibronectin 1	CAGCAGTACGGCCACCAAGA	ACGGTGTTGCTGGTCACAGG
FOXO3	Forkhead box O3	TCGAGAGCTCCCCGGACAAA	GGATGGAGTTCTTCCAGCCG
GCLC	Glutamate-cysteine ligase catalytic subunit	TTCACACTGCCCGAGGTCAA	CTTGTTAAGGTACTGAAGCGAGGGT
HAS3	Hyaluronan synthase 3	ACCAACCGAGTCCTGAGCCT	TGGTTGAGCCACCGGAGGTA
IVL	Involucrin	CCAGGCCAGGTCCAAGACAT	CTGGACACTGCGGGTGGTTA
KRT1	Keratin 1	TGACAAGGTGAGGTTCCTGGAG	AGTTGGTCCACTCTCCTTCGG
KRT10	Keratin 10	AGTCCCAACTGGCCTTGAAAC	AGCTGCACACAGTAGCGACC
KRT14	Keratin 14	TGCCGAGGAATGGTTCTTCACC	GCAGCTCAATCTCCAGGTTCTG
KRT5	Keratin 5	GCTGCCTACATGAACAAGGTGG	ATGGAGAGGACCACTGAGGTGT
LAMA5	Laminin subunit alpha 5	GAGGCTTTGAACCGGGCAGT	TCCTGCTTCCTTTGCAGGGC
LOR	Loricrin cornified envelope precursor protein	GTCTGCGGAGGTGGTTCCTCT	TGCTGGGTCTGGTGGCAGATC
MMP2	Matrix metallopeptidase 2	TGAGGGCGCTCTGTCTCCTG	TCAGGTATTGCACTGCCAACTC
NQO 1	NAD(P)H quinone dehydrogenase 1	CCTGCCATTCTGAAAGGCTGGT	GTGGTGATGGAAAGCACTGCCT
Nrf2	Nuclear factor, erythroid 2 like 2	CCAACTACTCCCAGGTTGCCC	GTGACTGAAACGTAGCCGAAGA
OCLN	Occludin	ATGGCAAAGTGAATGACAAGCGG	CTGTAACGAGGCTGCCTGAAGT
SIRT3	Sirtuin 3	CCACCTGCACAGTCTGCCAA	CAGCGGCTCCCCAAAGAACA
SOD1	Superoxide dismutase 1	GCACACTGGTGGTCCATGAAA	CAAGCCAAACGACTTCCAGCG
SOD2	Superoxide dismutase 2	CCCAAAGGGGAGTTGCTGGAA	CCAAGCCAACCCCAACCTGA
SPTLC1	Serine palmitoyltransferase long chain base subunit 1	GCAGTGTTGAAGGAAAAGTGCGG	CAGTGCTCTCTTCCAGTTGTAGG
TGFB1	Transforming growth factor beta 1	GTTGTGCGGCAGTGGTTGAG	CCGGTAGTGAACCCGTTGATG
TGM1	Transglutaminase 1	GAACGACTGCTGGATGAAGAGG	CTTGATGGACTCCACAGAGCAG
ZO1	Zonula occludens-1	GTCCAGAATCTCGGAAAAGTGCC	CTTTCAGCGCACCATACCAACC

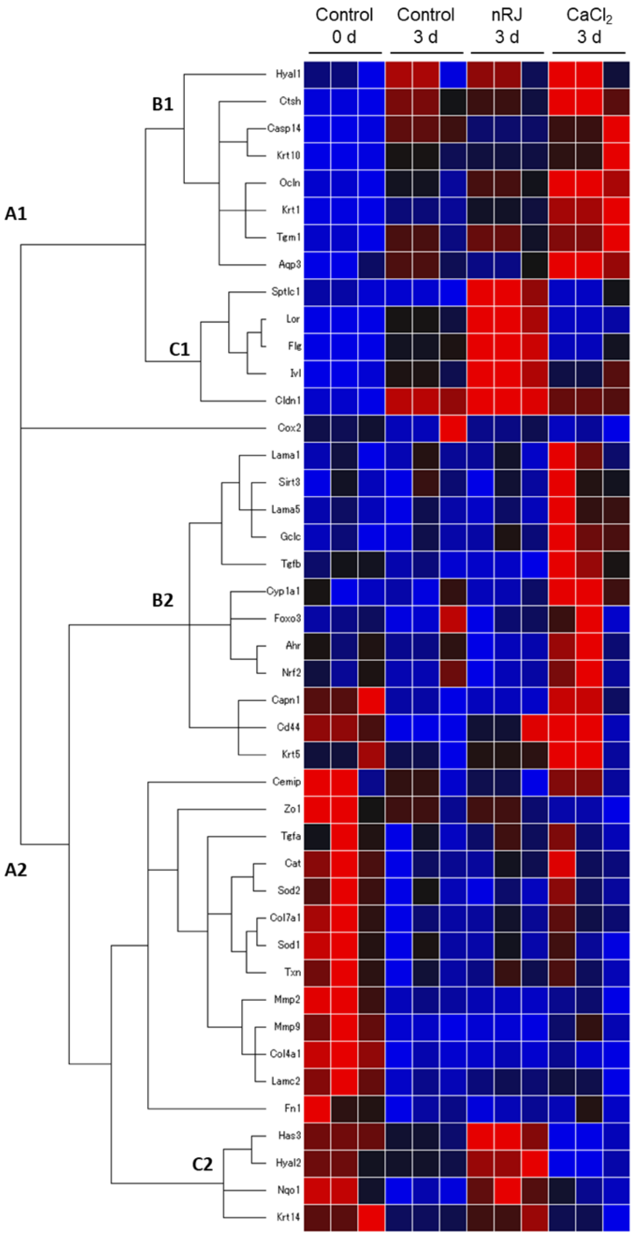

Supplemental Figure S1. Comparison of the gene expression pattern in keratinocytes following stimulation with RJ or keratinocyte differentiation inducer, $CaCl_2$. Two major clusters, A1 and A2, were observed before (0 d) and after (3 d) stimulation. With CaCl2, there were two clusters, B1 and B2. B1 contained the keratinocyte differentiation markers *Krt1* and *Krt10*. On the other hand, nRJ-treated keratinocytes expressed another characteristic of gene expression that was classified into clusters C1 and C2. In cluster C1, both stratum spinosum and granular layer related genes such as *Flg* and *Lvl* were observed.

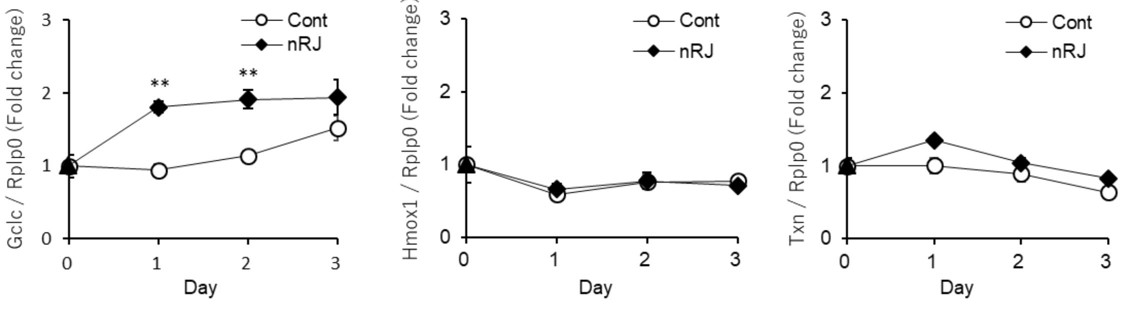

Supplemental Figure S2. Changes in Nrf2 downstream genes following stimulation with nRJ

Keratinocytes (pooled) were treated with 1 mg/ml nRJ for 3 days. Expression of *Gclc, Hmox1,* and *Txn* was analyzed using real-time PCR. Data represent mean ± SEM, n = 3-4, **p < 0.01, vs. Control (Cont), Student's *t*-test.

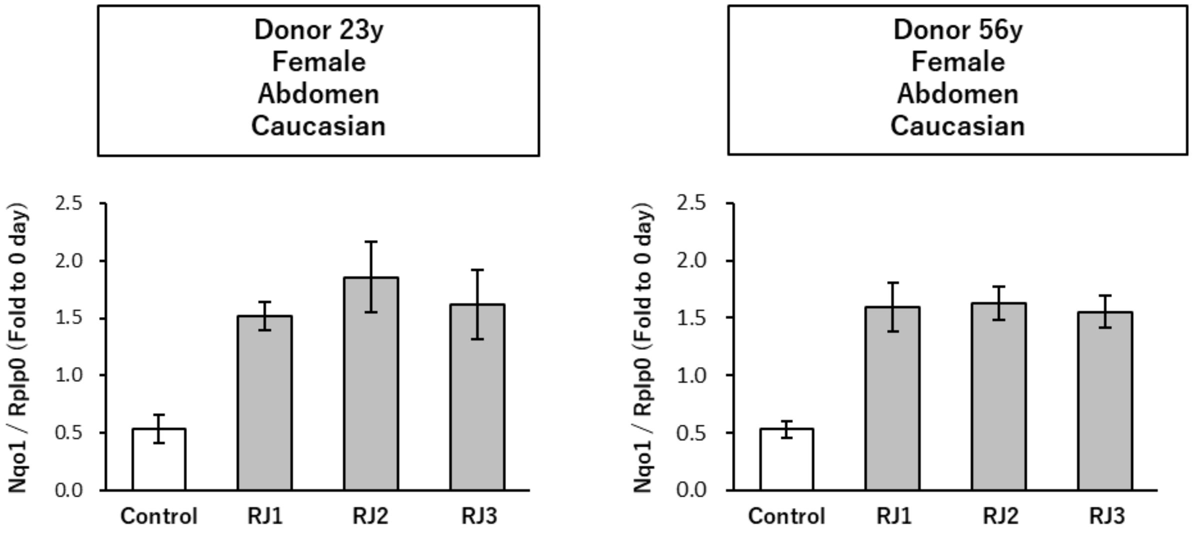

Supplemental Figure S3. Induction of NQO1 expression in keratinocytes (derived from 23y donor and 56 y donor) was comparable among different lots of nRJ.

Keratinocytes were treated with 1 mg/ml nRJ (RJ1 Lot YDP-M-180120, RJ2 Lot YDP-M-190801, and RJ3 Lot YDP-M-200225) for 3 days. NQO1 mRNA expression was analyzed by real-time PCR. Data represent the mean ± SEM, n=3.
